# Targeting the mitochondrial protein YME1L to inhibit osteosarcoma cell growth in vitro and in vivo

**DOI:** 10.1038/s41419-024-06722-6

**Published:** 2024-05-20

**Authors:** Xu Sun, Ce Shi, Jin Dai, Mei-Qing Zhang, Dong-Sheng Pei, Lei Yang

**Affiliations:** 1 Department of Hand and Foot Surgery, The Affiliated Taizhou People’s Hospital of Nanjing Medical University, Taizhou School of Clinical Medicine, Nanjing Medical University, Taizhou, China; 2 Cancer Institute, Xuzhou Medical University, Xuzhou, Jiangsu China; 3 Department of Orthopedics, The Affiliated Suqian Hospital of Xuzhou Medical University, Suqian, China; 4 Department of Orthopedics, Suzhou Wujiang District Children’s Hospital, Suzhou, China; 5 Medical School, Soochow University, Suzhou, China; 6 Department of Orthopedics, Wujin Hospital Affiliated with Jiangsu University, Changzhou, China

**Keywords:** Bone cancer, Targeted therapies

## Abstract

Exploring novel diagnostic and therapeutic biomarkers is extremely important for osteosarcoma. YME1 Like 1 ATPase (YME1L), locating in the mitochondrial inner membrane, is key in regulating mitochondrial plasticity and metabolic activity. Its expression and potential functions in osteosarcoma are studied in the present study. We show that *YME1L* mRNA and protein expression is significantly elevated in osteosarcoma tissues derived from different human patients. Moreover, its expression is upregulated in various primary and immortalized osteosarcoma cells. The Cancer Genome Atlas database results revealed that *YME1L* overexpression was correlated with poor overall survival and poor disease-specific survival in sarcoma patients. In primary and immortalized osteosarcoma cells, silencing of YME1L through lentiviral shRNA robustly inhibited cell viability, proliferation, and migration. Moreover, cell cycle arrest and apoptosis were detected in YME1L-silenced osteosarcoma cells. YME1L silencing impaired mitochondrial functions in osteosarcoma cells, causing mitochondrial depolarization, oxidative injury, lipid peroxidation and DNA damage as well as mitochondrial respiratory chain complex I activity inhibition and ATP depletion. Contrarily, forced YME1L overexpression exerted pro-cancerous activity and strengthened primary osteosarcoma cell proliferation and migration. YME1L is important for Akt-S6K activation in osteosarcoma cells. Phosphorylation of Akt and S6K was inhibited after YME1L silencing in primary osteosarcoma cells, but was strengthened with YME1L overexpression. Restoring Akt-mTOR activation by S473D constitutively active Akt1 mitigated YME1L shRNA-induced anti-osteosarcoma cell activity. Lastly, intratumoral injection of YME1L shRNA adeno-associated virus inhibited subcutaneous osteosarcoma xenograft growth in nude mice. YME1L depletion, mitochondrial dysfunction, oxidative injury, Akt-S6K inactivation, and apoptosis were detected in YME1L shRNA-treated osteosarcoma xenografts. Together, overexpressed YME1L promotes osteosarcoma cell growth, possibly by maintaining mitochondrial function and Akt-mTOR activation.

## Introduction

Osteosarcoma (OS) is a common malignancy in the bone among children and teenagers [1, 2]. The current clinical treatments, including chemotherapy (doxorubicin, cisplatin and methotrexate etc), molecularly-targeted therapy, radiotherapy and tumor-resection surgery [3–5], have been able to significantly improve the prognosis of patients with OS [6]. However, for the patients with the recurrent, metastatic, and other advanced tumors, the 5 year overall survival is still low (close to 20%) [2, 7]. It is therefore urgent to explore novel molecular targets vital for the oncogenesis and tumorigenesis of OS [8–10].

Mitochondria are the main organelle for energy metabolism, ATP synthesis and macromolecules biosynthesis [11, 12]. Elevated mitochondrial bioenergetics is required for the growth of OS cells and the progression of the tumor [13–15]. A number of key mitochondrial proteins are required for maintaining mitochondrial functions and the hyper-proliferative activity of OS cells. For example, Zhuo et al. recently reported that the mitochondrial protein ADCK1 (AarF domain-containing kinase 1) is overexpressed in OS and is required for OS cell in vitro and in situ growth [16]. Han et al. reported that the mitochondrial protein POLRMT (RNA polymerase mitochondrial) is overexpressed OS tissues and cells [17]. Contrarily, POLRMT silencing or knockout (KO) resulted in significant anti-OS cell activity [17].

The inner mitochondrial membrane-locating YME1L (YME1 Like 1 ATPase) is a primary member of AAA family ATPase proteins [18–24]. YME1L is important for the mitochondrial functions, morphology, plasticity, among others [18–20]. In inner mitochondrial membrane, YME1L is assembled into a homo-oligomeric complex [20–24]. The AAA family ATPase also regulates degradation of key mitochondrial proteins, including lipid-transferring proteins, inner mitochondrial translocation proteins, and the dynamin-like GTPase optic atrophy 1 (OPA1) [20–22, 25]. YME1L silencing decreased HEK-293 cell proliferation and altered the morphology of cristae [23]. YME1L depletion also induced oxidative stress and impaired cell respiration [23]. YME1L knockdown led to accumulation of non-assembled respiratory chain subunits, such as ND1, Ndufb6, and Cox4 [23].

Recent studies have proposed a pivotal role of YME1L in cancer cells. A recent study by Xia et al. reported that expression of YME1L was increased in non-small cell lung cancer (NSCLC) tissues/cells and its overexpression was required for NSCLC cell growth [26]. YME1L shRNA or dCas9/sgRNA-induced YME1L knockout (KO) robustly inhibited NSCLC cell growth in vitro and in vivo [26]. Liu et al. recently reported upregulation of YME1L in human glioma tissues and cells, which promoted in vitro and intracranial growth of glioma cells [27]. The mitochondrial protein YME1L is also essential for the growth of pancreatic ductal adenocarcinoma (PDAC) cells [19]. Its expression and potential functions in OS have not been studied thus far.

## Materials and methods

### Reagents and antibodies

Fluorescence probes, including TUNEL, tetraethylbenzimidazolylcarbocyanine iodide (JC*-*1), DAPI (4’,6-diamidino-2-phenylindole), CellROX, dichlorodihydrofluorescein diacetate (DCF-DA), EdU, Annexin V, propidium iodide (PI) were obtained from Thermo-Fisher Invitrogen (Shanghai, China). All antibodies and mRNA primers were provided by Dr. Cao at Soochow University [27]. Fetal bovine serum (FBS), high-glucose medium and antibiotics were provided by Hyclone (Logan, UT). Puromycin, polybrene and other chemicals were obtained from Sigma-Aldrich Chemicals Co. (St. Louis, MO).

### Cells

The primary human OS cells that were derived from three different patients, namely “pOS-1”, “pOS-2” and “pOS-3”, as well as the immortalized U2OS cells were provided by Dr. Ling [16]. The primary human osteoblasts (“pOB”) and hFOB1.19 osteoblastic cells were reported in our previous study [28]. The protocols of using human cells were approved the Ethic Committee of Taizhou People’s Hospital, according with the principles expressed in the Declaration of Helsinki.

### Human tissues

OS tumor tissues and the matched adjacent normal bone tissues from a total of sixteen (16) written-informed consent primary OS patients were provided by Dr. Ling at Soochow University [16]. Another ten pairs of OS tissues and matched adjacent normal tissues were from locally treated primary OS patients from our institution. A total of 26 pairs of tissues were obtained. All patients provided the written-informed consent. Protocols of using human tissues were approved by the Ethic Committee of Taizhou People’s Hospital, in according to the principles of the Declaration of Helsinki.

### Quantitative real time-PCR (qRT-PCR)

As reported [29, 30], total cellular RNA was extracted by the TRIzol reagents and was reversely transcripted [31]. The qRT-PCR was performed through the ABI Prism 7900 system under the SYBR GREEN PCR Master Mix (Thermo-Fisher Scientific). The product melting temperature was always calculated. *Glyceraldehyde-3-phosphatedehydrogenase* (*GAPDH)* was examined as the internal control for data quantification (2^−∆∆*C*t^ method).

### Western blotting

In brief, protein lysates were separated by SDS-PAGE and were transferred to polyvinylidene fluoride (PVDF) blots. The blots were blocked (in 7.5% milk PBST solution) and were incubated with indicated primary and secondary antibodies. To visualize the targeted protein bands, the chemiluminescence (ECL) reagent kit (Bio-Rad, Shanghai, China) was applied. Data quantification was through the ImageJ software (NIH). Supplementary Fig. S1 contained the uncropped blotting images of the study.

### YME1L shRNA

The lentivirus (using GV369 construct, Genechem) encoding two different shRNAs (shYME1L-seq1 and shYME1L-seq2) against human *YME1L* were provided by Dr. Cao at Soochow University [27]. The primary/established OS cells, the human osteoblasts or hFOB1.19 osteoblastic cells were cultivated in polybrene-containing complete medium and were treated with the lentivirus (at MOI = 15) for 48 h. Cells were then maintained under puromycin-containing medium for another 8–10 days and stable cells were formed. Expression of YME1L was always examined by qRT-PCR and Western blotting analyses. The YME1L shRNA-expressing adeno-associated virus (AAV) or shC-expressing AAV was from Dr. Zha [26].

### YME1L overexpression

The lentivirus (using GV369 construct, Genechem) encoding the YME1L-expressing sequence was also from Dr. Cao [27]. The primary human OS cells were cultivated in polybrene-containing complete medium and were infected with the YME1L-expressing lentivirus (at MOI = 15) for 72 h. Cells were then maintained under puromycin-containing medium for another 8–10 days and stable cells were formed. Expression of YME1L was verified by qRT-PCR and western blotting analyses.

### EdU (5-Ethynyl-20-deoxyuridine) staining

The Apollo-567 EdU incorporation Kit (RiboBio, Guangzhou, China) was utilized. Cells were seeded into 24-well plates (at 5 × 10^4^ cells per well) for indicated durations and stained with 30 mM EdU for 2 h before fixation with 4% paraformaldehyde, while nuclei were stained with DAPI for 20 min. Visualization of EdU-positive nuclei was through ZEISS LSM fluorescence microscope. At least five randomly chosen fields were analyzed for the EdU ratio (EdU/DAPI×100%) under each condition.

### TUNEL (TdT-mediated dUTP nick-end labeling) staining

One-step TUNEL Cell Apoptosis Detection Kit (Beyotime Biotechnology, Shanghai, China) was utilized. Cells were seeded into 24-well plates (at 5 × 10^4^ cells per well) for indicated periods. After fixing cells in 4% paraformaldehyde for 30 min and treating them with Triton for 5 min, cells were incubated with TUNEL detection solution at 37 °C for 1 h, followed by DAPI nuclei staining for 20 min. Visualization of TUNEL-positive nuclei was through ZEISS LSM fluorescence microscope. At least five randomly chosen fields were analyzed for the TUNEL ratio (TUNEL/DAPI×100%) under each condition.

### Fluorescence staining in cells

Cells were seeded into 12-well plates (at 5 × 10^4^ cells per well). Following treatment and culturing, cells were stained with specific fluorescence dyes (JC-1, DCF-DA and CellROX) in staining buffer for 30 min at 37 °C in a CO_2_ incubator, ensuring protection of cells from light during staining. Following incubation, cells were washed with PBS. The fluorescence intensity was measured by a Thermo Scientific Fluoroskan Microplate Fluorometer at the designated excitation/emission wavelengths (in the attached protocols), with the fluorescence intensity density recorded and quantified. The fluorescence images were also photographed under an ZEISS LSM fluorescence microscope.

### Cell migration and invasion assays

“Transwell” chambers (Corning, Shanghai, China) were utilized. Cells carrying specific genetic alterations were suspended in serum-free medium and placed on the chamber surface. The lower chamber was filled with medium containing 10% FBS. Following a 24-h incubation, upper-surface cells were gently removed, while the lower chamber membranes were fixed using 4% paraformaldehyde and stained with crystal violet. For in vitro cell invasion assays, Matrigel (Sigma) pre-coating of “Transwell” chambers was performed before initiating the experiments.

### FACS

The cells subjected to designated genetic treatments were cultured for described duration and were trypsinized and re-suspended. Cells were then labeled with Annexin V-PE (10 μg/mL) and/or propidium iodide (PI) (10 μg/mL, Calbiochem, China). Subsequently, cells underwent detection through FACS analysis using a Becton-Dickinson Flow Cytometer (BD Bioscience, Shanghai, China).

### Other cellular functional studies

The cell-counting-kit-8 (CCK-8) cell viability, trypan blue assaying of cell death, Caspase-3/Caspase-9 activity assays, single strand DNA (ssDNA) ELISA were described in detail in our previous studies [28, 30, 32, 33]. The protocols of lipid peroxidation detection by measuring thiobarbituric acid reactive substances (TBAR) were described in other studies [34, 35]. ATP contents in cellular and tissue lysates as well as the mitochondrial respiratory chain complex I (mito-complex I) activity were measured as previously described [36, 37].

### Constitutively active Akt1 or Gαi1 overexpression

The lentiviral particles encoding the S473D constitutively active mutant Akt1 (caAkt1) were from Dr. Xu’s group [34] and were added to pOS-1 primary cells. Following selection by puromycin stable cells were formed [34]. The lentiviral Gαi1-overexpressing construct or the empty vector, from Dr. Cao [38, 39], was added to primary OS cells, and stable cells established after puromycin selection.

### OS xenograft studies

The animal procedures were approved by the Ethics Committee and Animal Care Committee of Taizhou People’s Hospital and were described in our previous study [28]. The 5–6 week-old BALB/c nude mice (half male half female, 18.1–18.5 g in weights) were provided by Shanghai Slake Laboratory Animal Co. (Shanghai, China). Mice were maintained under the standard condition [28]. The pOS-1 primary OS cells (at seven million cells in each mouse), as reported [28], were subcutaneously (s.c.) injected into the flanks of nude mice. The OS xenografts were thereafter established after 20 days and were subject to the described treatments. Tumor volumes were calculated by the described formula [28]. The estimated daily tumor growth and animal body weights were also recorded [28]. For tissue immunofluorescence studies, the sections were stained with TUNEL and counterstained with DAPI, washed twice, and visualized under a confocal microscope.

### Statistical analyses

In vitro experiments were repeated five times. Data were with normal distributions and were presented as mean ± standard deviation (SD). The difference between groups was determined by the two-tailed Student’s *t* test (Excel 2010, for two specific groups) or ANOVA analysis with Student–Newman–Keuls post hoc test (SPSS 23.0, for multiple groups). *P* values < 0.05 were statistically significant.

## Results

### YME1L upregulation in human OS tissues and cells

**Fig. 1:**
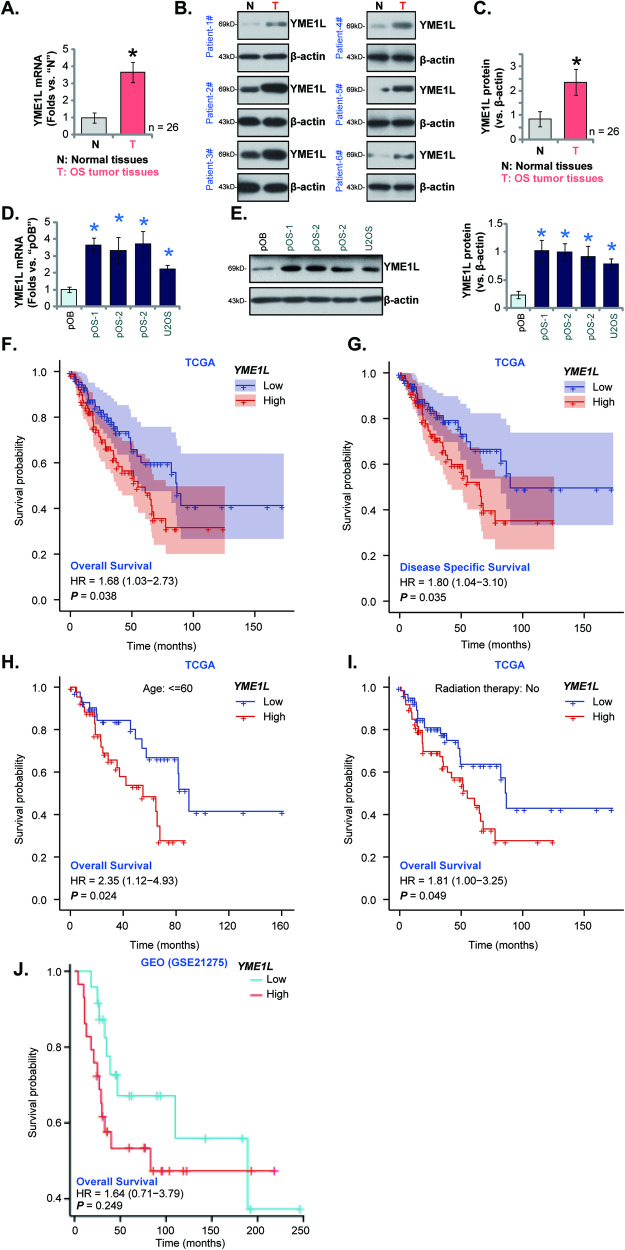
YME1L upregulation in human OS tissues and cells. Expression of *YME1L* mRNA (**A**) and protein (**B**, **C**) in the OS tissues (“T”) and matched adjacent normal tissues (“N”) from 26 different primary OS patients is shown; Expression of *YME1L* mRNA (**D**) and protein (**E**) in the listed OS cells and primary human osteoblasts (“pOB”) is shown. Expression of *YME1L* mRNA in The Cancer Genome Atlas (TCGA) sarcoma tissue cohort was retrieved, and Kaplan–Meier plus univariate Cox survival analyses showed the correlation between *YME1L* expression and patients’ overall survival (**F**) or patients’ disease-free survival (**G**). Kaplan–Meier plus univariate Cox survival analyses showed the correlation between *YME1L* expression and overall survival in sarcoma patients age <=60 (**H**), or in sarcoma patients with no radiation therapy (**I**). Gene Expression Omnibus (GEO) GSE21275 OS dataset showed the correlation between *YME1L* expression and patient survival (**J**). Error bars stand for mean ± standard deviation (SD). *P < 0.001 versus “N” tissues or “pOB” cells.

First, we tested YME1L expression in sixteen (*n* = 26) different OS tissues (“T”) and matched adjacent normal tissues (“N”)-derived from primary patients. As shown, *YME1L* mRNA expression in the OS tissues was significantly higher than that in the adjacent normal tissues (Fig. 1A). Moreover, elevated YME1L protein expression was detected in OS tissues of six representative patients (“Patient-1” to “Patient-6”) (Fig. 1B). After combining all 16 pairs of tissue data, we found that YME1L protein upregulation in human OS tissues was significant (*P* < 0.001 vs. normal tissues, Fig. 1C).

Expression of YME1L in OS cells was examined as well. The primary OS cells derived from three patients, “pOS-1”, “pOS-2”, and “pOS-3”, as well as the immortalized U2OS cells, were cultured. *YME1L* mRNA expression in the OS cells was significantly higher than that in the primary human osteoblasts (“pOB”) (Fig. 1D). Moreover, YME1L protein levels were also upregulated in the primary and immortalized OS cells (Fig. 1E), and relatively low expression detected in pOB cells (Fig. 1E).

We also tested whether YME1L expression was correlated with clinical parameters of 252 sarcoma patients that were retrieved from The Cancer Genome Atlas (TCGA) database. Kaplan–Meier plus univariate Cox survival analyses showed that *YME1L* overexpression was significantly correlated with the poor overall survival [hazard ratio (HR): 1.68, *P* = 0.038] (Fig. 1F) and the poor disease-specific survival [DSS, hazard ratio (HR): 1.80, *P* = 0.035] (Fig. 1G) of the sarcoma patients. Further subgroup analyses revealed that high *YME1L* expression in sarcoma tissues was correlated with poor prognosis in sarcoma patients age <=60 ([hazard ratio (HR): 2.35, *P* = 0.024], Fig. 1H). In addition, for sarcoma patients with no radiation therapy, high *YME1L* expression also predicted poor prognosis ([hazard ratio (HR): 1.81, *P* = 0.049], Fig. 1I). Kaplan–Meier survival analysis and univariate Cox regression analysis of Gene Expression Omnibus (GEO) GSE21275 OS dataset also showed a potential correlation between YME1L overexpression and poor overall survival in OS patients, but lacking the statistical significance (*P* = 0.249) (Fig. 1J).

### YME1L co-expressed genes are enriched in cancer-related cascades in human OS tissues

**Fig. 2:**
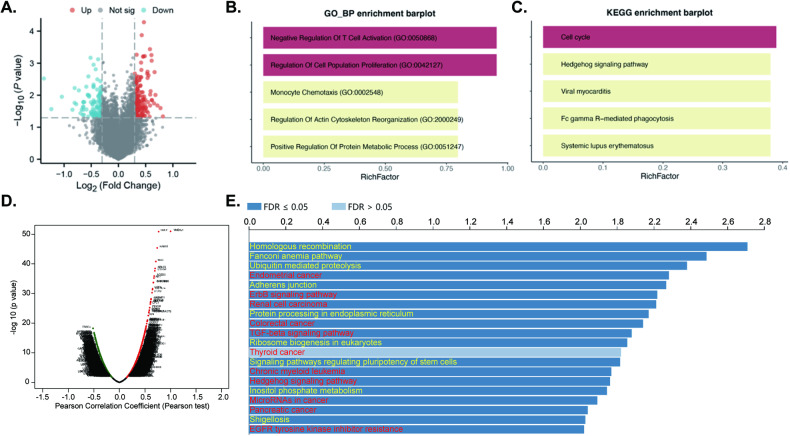
*YME1L* co-expressed genes are enriched in cancer-related cascades in human OS tissues. Therapeutically Applicable Research to Generate Effective Treatments (TARGET) database OS cohort demonstrated *YME1L*-co-expressing genes (CEGs) in OS tissues (**A**). The top 20 enriched biological processes (BP), via GO assay (**B**), and top enriched cascades, through KEGG analyses, are shown (**C**). The Cancer Genome Atlas (TCGA) sarcoma cohorts and LinkedOmics functional assays demonstrated *YME1L*-co-expressing genes in sarcoma tissues (**D**). The top 20 enriched pathways, through KEGG analyses, are shown (**E**).

The utilization of the Therapeutically Applicable Research to Generate Effective Treatments (TARGET) database enabled an extensive exploration of the possible function role of *YME1L* in OS. The differential analysis between high and low expression groups of *YME1L* identified 287 differentially expressed genes (DEGs) (Fig. 2A), demonstrating associations with “Negative Regulation Of T Cell Activation”, “Regulation Of Cell Population Proliferation”, and “Regulation of Actin Cytoskeleton Reorganization”, revealed through GO analysis (Fig. 2B). The KEGG signaling analyses indicated involvement of *YME1L*-dependent DEGs in “Cell Cycle”, “Hedgehog Signaling”, “Viral Myocarditis”, among others in OS (Fig. 2C).

Next, the mRNA sequencing data of 252 sarcoma patients in the TCGA database were retrieved and were analyzed through a LinkedOmics functional module by the described method [16]. The volcano plot, Fig. 2D, showed that the genes in red dots were positively correlated with *YME1L* expression in sarcoma tissues, whereas genes in green dots were negatively correlated with *YME1L* expression (false discovery rate [FDR] <0.01). Significant KEGG term annotation by overrepresentation enrichment analysis (ORA) showed the top twenty (20) pathways that were enriched from the *YME1L*-co-expressing genes (Fig. 2E). The majority of these pathways are vital for cancer progression, including “Endometrial cancer”, “Renal cell carcinoma”, “ErbB signaling pathway”, “Colorectal cancer”, “Thyroid cancer”, “Thyroid cancer” (Fig. 2E). These findings collectively suggest the possible multifaceted involvement of *YME1L* in regulating diverse signaling pathways and biological processes crucial to OS pathogenesis.

### YME1L silencing inhibits OS cell survival, proliferation, and migration

**Fig. 3:**
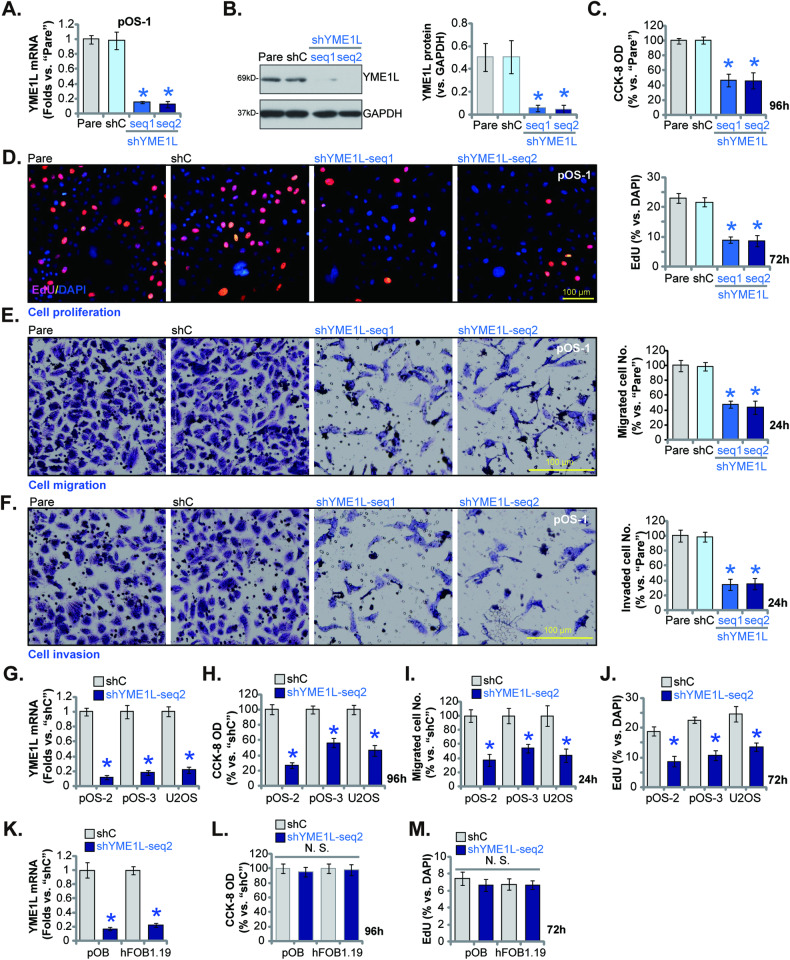
YME1L silencing inhibits OS cell survival, proliferation and migration. The patient-derived primary human OS cells (pOS-1), with the lentiviral YME1L shRNA (“shYME1L-seq1/shYME1L-seq2”, representing two different shRNAs) or scramble control shRNA (“shC”), were established and expression of *YME1L* mRNA (**A**) and protein (**B**) was examined. After cultivation for the indicated time periods, cell viability (**C**), proliferation (**D**), migration (**E**), and invasion (**F**) were tested using the described methods. The primary OS cells that were derived two other patients, pOS-2 and pOS-3 (**G**–**J**), the immortalized U2OS cells (**G**–**J**), the primary human osteoblasts (“pOB”) (**K**–**M**) or hFOB1.19 osteoblastic cells (**K**–**M**), with lentiviral shYME1L-seq2 or shC, were established and expression of *YME1L* mRNA was tested (**G**, **K**). After cultivation for the indicated time periods, cell viability (**H**, **L**), proliferation (**I**, **M**) and migration (**J**) were tested. Data were presented as mean ± standard deviation (SD, *n* = 5). *P < 0.001 versus “shC” group. “N. S.” indicated no statistical difference (*P* > 0.05). Each single experiment was repeated for five times. Scale bar = 100 μm.

Next experiments were carried out to examine whether overexpressed YME1L was important for cancerous behaviors of OS cells. The shRNA strategy was utilized to silence YME1L. Specifically, to the patient-derived primary human OS cells, pOS-1 [28], the lentivirus encoding the YME1L shRNA sequence (“shYME1L-seq1/shYME1L-seq2”, representing two different shRNAs) was added. Stable cells were thereafter established using the puromycin selection medium. *YME1L* mRNA (Fig. 3A) and protein (Fig. 3B) expression levels were robustly decreased in the stable pOS-1 cells with shYME1L-seq1/shYME1L-seq2. As shown, shRNA-induced silencing of YME1L resulted in significant viability (CCK-8 OD) reduction in pOS-1 cells (Fig. 3C). Moreover, YME1L shRNA suppressed pOS-1 cell proliferation and hindered nuclear EdU incorporation (Fig. 3D). Next, “Transwell” and “Matrigel Transwell” assays were carried out to examined the potential role of YME1L on cell mobility. Results demonstrated the two applied YME1L shRNAs potently slowed pOS-1 cell in vitro migration and invasion (Fig. 3E, F). The scramble control shRNA (“shC”), expectably, failed to alter YME1L expression (Fig. 3A, B) and pOS-1 cell functions (Fig. 3C–F).

In other OS cells whether YME1L silencing could result in similar anti-cancer activity was studied next. Specially, the primary OS cells that were derived two other patients, pOS-2 and pOS-3, and the immortalized U2OS cells were infected with shYME1L-seq2-expressing lentivirus, and stable cells formed with selection by puromycin. shYME1L-seq2 resulted in dramatic *YME1L* mRNA downregulation in the primary/established OS cells (Fig. 3G). Importantly, YME1L shRNA led to robust viability reduction in the OS cells (Fig. 3H). Moreover, cell proliferation (EdU ratio, Fig. 3I) and migration (Fig. 3J) were significantly inhibited following YME1L silencing in the primary and immortalized OS cells.

To the primary human osteoblasts (“pOB”) and hFOB1.19 osteoblastic cells, shYME1L-seq2-expressing lentivirus was added. Following selection, stable cells were formed, showing significantly decreased *YME1L* mRNA expression (Fig. 3K). Unlike the functional consequence in the OS cells, YME1L silencing failed to significantly decrease CCK-8 viability (Fig. 3L) and inhibit proliferation (EdU ratio reduction, Fig. 3M) in pOB and hFOB1.19 cells. These results supported YME1L silencing resulted in specific inhibition in OS cells.

### YME1L silencing induces cell cycle arrest and apoptosis in OS cells

**Fig. 4:**
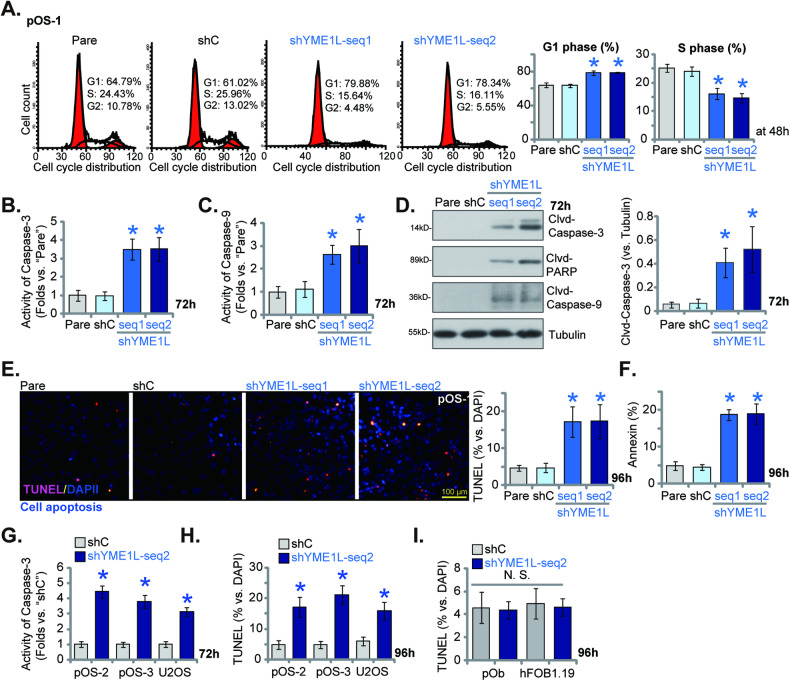
YME1L silencing induces cell cycle arrest and apoptosis in OS cells. The patient-derived primary human OS cells (pOS-1), with the lentiviral YME1L shRNA (“shYME1L-seq1/shYME1L-seq2”, representing two different shRNAs) or scramble control shRNA (“shC”), were established and cultivated for the indicated time periods, cell cycle distribution was examined (**A**). Caspase-PARP activation was analyzed (**B**–**D**), with cell apoptosis examined by nuclear TUNEL staining (**E**) and Annexin V FACS (**F**) assays. The primary OS cells that were derived two other patients, pOS-2 and pOS-3 (**G**, **H**), the immortalized U2OS cells (**G**, **H**), the primary human osteoblasts (“pOB”) (**I**) or hFOB1.19 osteoblastic cells (**I**), with lentiviral shYME1L-seq2 or shC, were cultivated for 72/96 h, the Caspase-3 activity (**G**) and apoptosis (nuclear TUNEL staining, **H**, **I**) were tested. Data were presented as mean ± standard deviation (SD, *n* = 5). *P < 0.001 versus “shC” group. Each single experiment was repeated for five times. Scale bar = 100 μm.

Mitochondrial metabolism and ATP production are essential for cell cycle progression in OS and other cancer cells [16, 17, 40]. We next examined whether YME1L silencing could disrupt cell cycle in OS cells. As shown, YME1L silencing, by the two different shRNA (shYME1L-seq1/shYME1L-seq2, see Fig. 3), led to G1-phase increasing but S-phase decreasing in pOS-1 primary cells (Fig. 4A). Thus, YME1L silencing resulted in G1-S arrest in OS cells. Cell cycle arrest and proliferation inhibition can induce apoptosis in OS cells [16, 17, 28]. In pOS-1 primary cells, shRNA-induced silencing of YME1L increased the activity of Caspase-3 (Fig. 4B) and Caspase-9 (Fig. 4C). Cleavages (“clvd”) of Caspase-3, PARP and Caspase-9 were strengthened in YME1L-silenced pOS-1 cells (Fig. 4D). Moreover, apoptosis activation was detected in YME1L shRNA-expressing pOS-1 cells, as the TUNEL-nuclei ratio (Fig. 4E) and Annexin V-positive staining cells (Fig. 4F) were both significantly increased in YME1L-silenced pOS-1 cells.

In other primary human OS cells (pOS-2 and pOS-3) and the immortalized U2OS cells, YME1L silencing by shYME1L-seq2 similarly increased Caspase-3 activity (Fig. 4G). The increasing of TUNEL-stained nuclei supported apoptosis activation in YME1L-silenced primary/established OS cells (Fig. 4H). While in the primary human osteoblasts (“pOB”) and hFOB1.19 osteoblastic cells, YME1L silencing by the same shYME1L-seq2 failed to induce apoptosis activation, as the TUNEL-positive nuclei ratio was not significantly altered (Fig. 4I). These results supported that YME1L silencing induced cell cycle arrest and apoptosis in OS cells.

### YME1L silencing impairs mitochondrial functions in OS cells

**Fig. 5:**
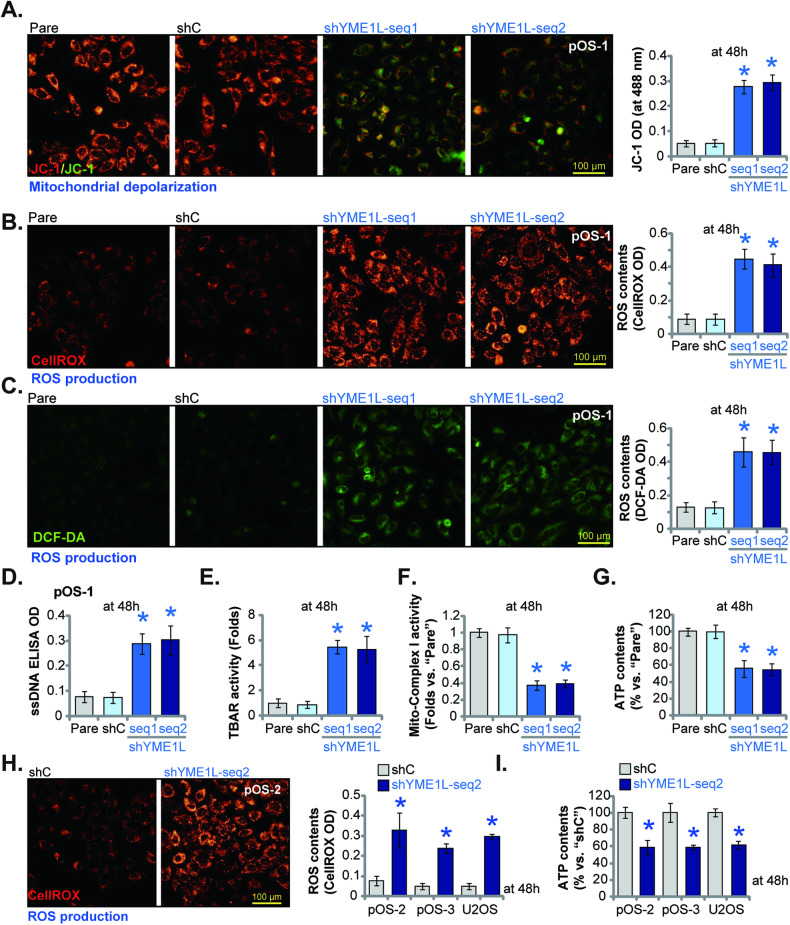
YME1L silencing impairs mitochondrial functions in OS cells. The patient-derived primary OS cells (pOS-1), with the lentiviral YME1L shRNA (“shYME1L-seq1/shYME1L-seq2”, representing two different shRNAs) or scramble control shRNA (“shC”), were established and cultivated for 48 h, mitochondrial depolarization (by testing JC-1 green monomers intensity, **A**), ROS production (by measuring CellROX/DCF-DA intensity, **B** and **C**), ssDNA contents (**D**) and lipid peroxidation (by measuring TBAR activity, **E**), as well as the mitochondrial respiratory chain complex I (mito-complex I) activity (**F**) and ATP contents (**G**) were measured. The primary OS cells that were derived two other patients, pOS-2 and pOS-3 or the immortalized U2OS cells, with lentiviral shYME1L-seq2 or shC, were cultivated for 48 h, ROS production (CellROX intensity, **H**) or ATP contents (**I**) were measured. Data were presented as mean ± standard deviation (SD, *n* = 5). *P < 0.001 versus “shC” group. Each single experiment was repeated for five times. Scale bar = 100 μm.

YME1L is a mitochondrial protein essential for maintaining mitochondrial functions [18, 20, 23, 27]. We thus analyzed the potential role of YME1L on the mitochondrial functions in OS cells. As shown, in the patient-derived primary OS cells (pOS-1), YME1L silencing by shYME1L-seq1/shYME1L-seq2 (see Figs. 3 and 4) led to mitochondrial depolarization. The latter was evidenced by the accumulation of JC-1 green monomers (Fig. 5A). Moreover, YME1L shRNA led to robust ROS production and oxidative injury in pOS-1 cells, as the CellROX red fluorescence intensity (Fig. 5B) and the DCF-DA green fluorescence intensity (Fig. 5C) were both significantly augmented. The applied YME1L shRNAs also induced DNA damage and increased ssDNA contents in pOS-1 cells (Fig. 5D). Moreover, the increased TBAR activity supported lipid peroxidation in pOS-1 cells following YME1L silencing (Fig. 5E). To further support mitochondrial function impairment, we found that the mitochondrial respiratory chain complex I (mito-complex I) activity was remarkably reduced following YME1L silencing in pOS-1 cells (Fig. 5F). Moreover, YME1L shRNA led to robust ATP reduction (Fig. 5G). In other primary OS cells (pOS-2 and pOS-3) and immortalized U2OS cells, YME1L silencing by shYME1L-seq2 (see Figs. 3 and 4) induced ROS production and caused CellROX fluorescence intensity increasing (Fig. 5H). ATP contents were however decreased in the YME1L-silenced OS cells (Fig. 5I). Together, these results showed that YME1L silencing impaired mitochondrial functions in OS cells.

### YME1L overexpression exerts pro-cancerous activity in OS cells

**Fig. 6:**
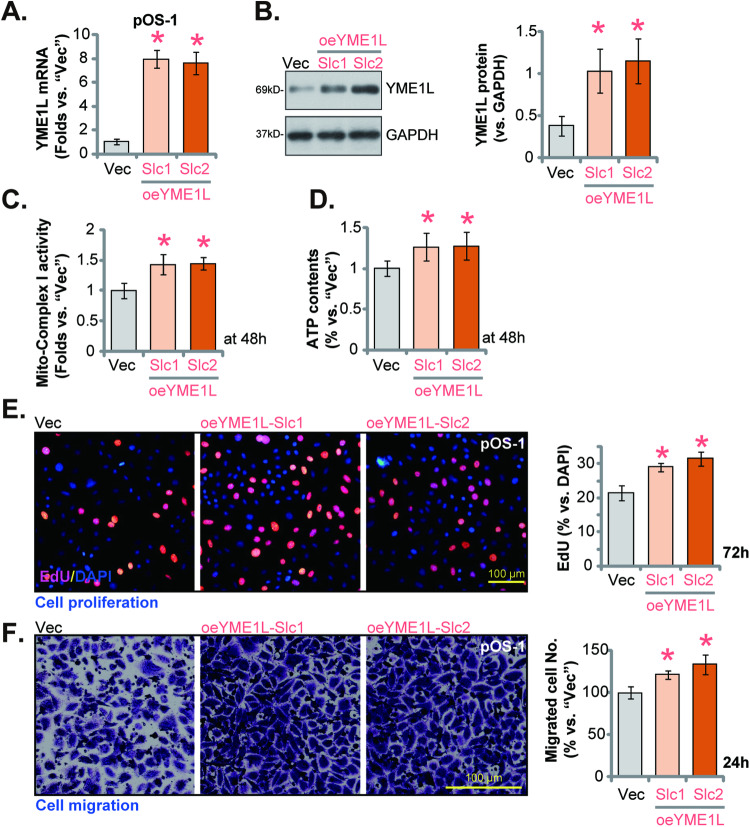
YME1L overexpression exerts pro-cancerous activity in OS cells. The patient-derived primary OS cells (pOS-1), with the lentiviral YME1L-overexpressing construct (“oeYME1L-Slc1/oeYME1L-Slc2”, representing two stable selections) or the empty vector (“Vec”), were established, and expression of *YME1L* mRNA (**A**) and protein (**B**) was examined. Cells were further and cultivated for indicated time periods, the mitochondrial respiratory chain complex I (mito-complex I) activity (**C**), ATP contents (**D**), cell proliferation (**E**), and migration (**F**) were tested by the described methods. Data were presented as mean ± standard deviation (SD, *n* = 5). *P < 0.001 versus “Vec” group. Each single experiment was repeated for five times. Scale bar = 100 μm.

Since YME1L depletion resulted in robust anti-cancer activity in OS cells, we hypothesized that ectopic overexpression of YME1L could possibly exert pro-cancerous activity. Thus, the lentivirus encoding YME1L-overexpressing construct (from Dr. Cao [26]) was added to cultured pOS-1 cells. Following puromycin treatment, two stable sections, oeYME1L-Slc1, and oeYME1L-Slc2, were achieved. In these cells, expression of *YME1L* mRNA (Fig. 6A) and protein (Fig. 6B) was dramatically elevated. Moreover, the mito-complex I activity was strengthened (Fig. 6C), and ATP contents were increased (Fig. 6D) in YME1L-overexpressing pOS-1 cells. Functional studies revealed that ectopic overexpression of YME1L promoted pOS-1 cell proliferation and facilitated nuclear EdU incorporation (Fig. 6E). Moreover, pOS-1 cell in vitro migration (Fig. 6F) was accelerated following YME1L overexpression. Therefore, YME1L overexpression exerted pro-cancerous activity in OS cells, further supporting the role of the mitochondrial protein in the progression of OS cells.

### YME1L is important for Akt-mTOR activation in OS cells

**Fig. 7:**
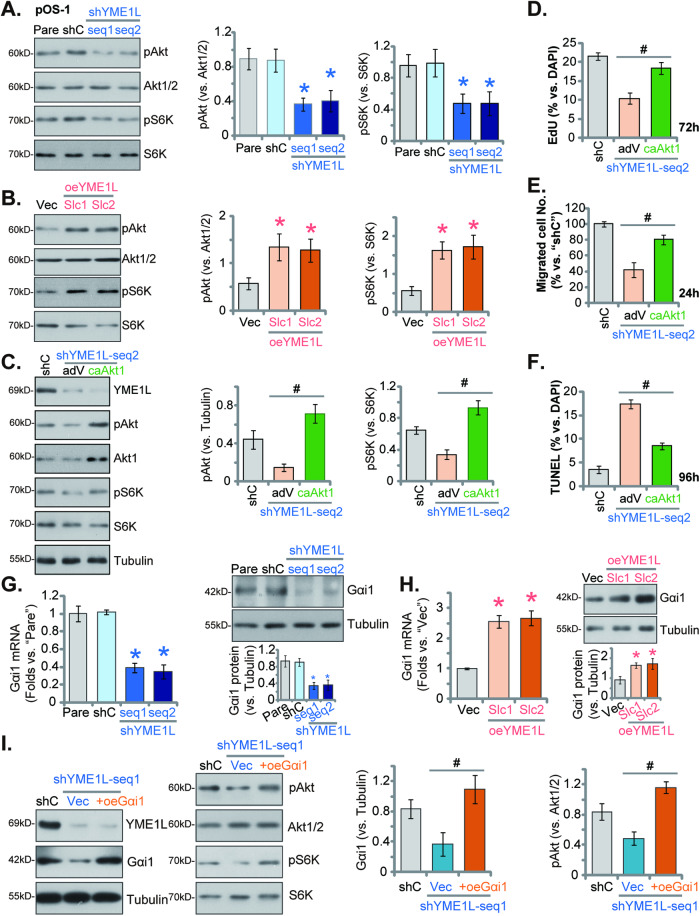
YME1L is important for Akt-mTOR activation in OS cells. Expression of the listed proteins in pOS-1 cells with the lentiviral YME1L shRNA (“shYME1L-seq1/shYME1L-seq2”, representing two different shRNAs), scramble control shRNA (“shC”), the lentiviral YME1L-overexpressing construct (“oeYME1L-Slc1/oeYME1L-Slc2”, representing two stable selections), or the empty vector (“Vec”) is shown (**A**, **B**, **G**, **H**). The shYME1L-seq2-expressing pOS-1 cells were further infected with adenovirus-encoded S473D constitutively active mutant Akt1 (caAkt1) or empty vector (“adV”), with stable cells formed after puromycin selection. Expression of the listed proteins is shown (**C**). After cultivation for the indicated time periods, cell proliferation, migration and apoptosis were examined by nuclear EdU staining (**D**), Transwell (**E**), and nuclear TUNEL staining (**F**) assays, respectively, with quantified results presented. The pOS-1 cells with shYME1L-seq1 were further transduced with a lentiviral Gαi1-expressing construct (“oeGαi1”) or the empty vector (“Vec”), and stable cells established after puromycin selection. Expression of listed proteins was tested (**I**). Data were presented as mean ± standard deviation (SD, *n* = 5). *P < 0.001 versus “shC”/”Vec” group. ^#^*P* < 0.001^.^ *“*N. S.” indicated no statistical difference (*P* > 0.05). Each single experiment was repeated for five times.

Recent studies have proposed that YME1L is important for Akt-mTOR cascade activation in glioma and NSCLC cells [26, 27, 37]. Considering the important role of Akt-mTOR cascade in the tumorigenesis and progression of OS [32, 41–43], we next tested whether genetic alteration of YME1L could alter Akt-mTOR signaling activation in OS cells. As shown, YME1L silencing, by shYME1L-seq1/shYME1L-seq2 (see Figs. 3–5), significantly inhibited phosphorylation of Akt (at Ser-473) and S6K (at Thr-389) in pOS-1 cells (Fig. 7A), leaving total Akt1/2 and S6K1 unaltered (Fig. 7A). These results supported that YME1L shRNA inhibited Akt-mTOR activation in OS cells. Contrarily, in YME1L-overexpressed pOS-1 cells, oeYME1L-Slc1 and oeYME1L-Slc2 (see Fig. 6), Akt (at Ser-473) and S6K (at Thr-389) phosphorylation were strengthened (Fig. 7B), and total Akt1/2 and S6K1 expression again unaltered (Fig. 7B).

We further hypothesized that Akt-mTOR inactivation could be one important mechanism of YME1L silencing-caused anti-OS cell activity. Thus, the S473D constitutively active mutant Akt1 (caAkt1)-expressing viral construct was stably transduced to shYME1L-seq2-expressing pOS-1 cells. After puromycin selection stable cells were established. The mutant caAkt1 completely restored phosphorylation of Akt (at Ser-473) and S6K (at Thr-389) in YME1L-silence pOS-1 cells (Fig. 7C), without alter YME1L protein expression (Fig. 7C). Importantly, quantified results showed that YME1L silencing (by shYME1L-seq2)-induced proliferation arrest (Fig. 7D), migration declining (Fig. 7E), and apoptosis induction (Fig. 7F) were mitigated, but not reversed, by the mutant Akt1. These results supported that Akt-mTOR inactivation is one important mechanism of YME1L depletion-caused anti-OS cell activity.

A previous study demonstrated the crucial role of YME1L within glioma cells in facilitating the transcription and expression of G protein subunit alpha i1 (Gαi1) [27], a key protein essential for the activation of Akt-mTOR cascade through various receptor tyrosine kinases (RTKs) and non-RTK receptors [38, 39, 44–51]. We revealed that the suppression of YME1L, achieved through shYME1L-seq1 or shYME1L-seq2, markedly downregulated both mRNA and protein levels of Gαi1 in pOS-1 primary cells (Fig. 7G). Conversely, the overexpression of YME1L in pOS-1 cells led to a significant increase in *Gαi1* mRNA and protein expression (see Fig. 7H). Crucially, in pOS-1 cells expressing shYME1L-seq1, the augmentation of Gαi1 expression via a lentiviral Gαi1-expressing construct (“oeGαi1”, from Dr. Cao [38, 39]) reinstated the phosphorylation of Akt and S6K (Fig. 7I). These findings supported that YME1L-promoted Akt-mTOR activation was possibly due to promoting Gαi1 expression in OS cells.

### Intratumoral injection of AAV-packed YME1L shRNA inhibits subcutaneous OS xenograft growth in nude mice

**Fig. 8:**
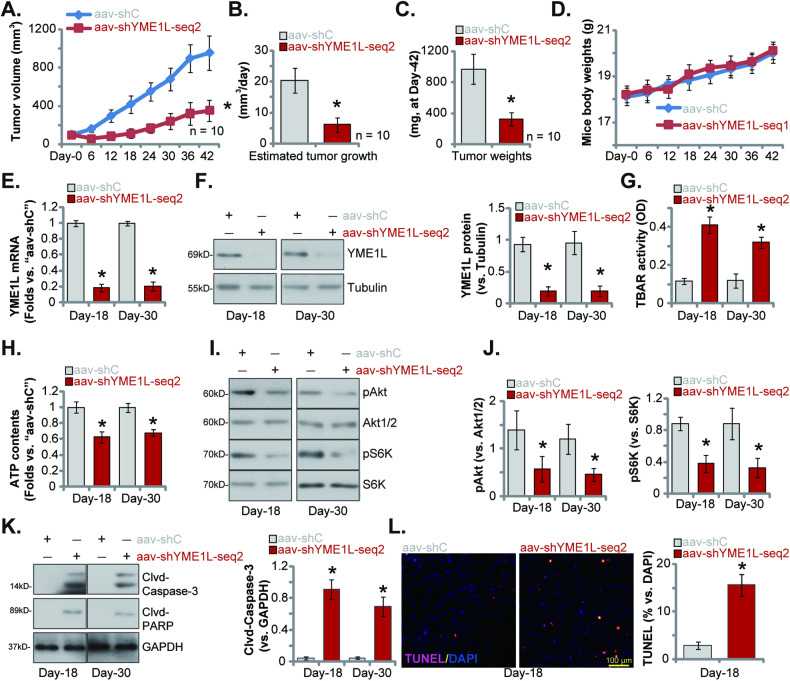
Intratumoral injection of AAV-packed YME1L shRNA inhibits subcutaneous OS xenograft growth in nude mice. The pOS-1 xenograft-bearing mice were intratumorally injected with AAV-packed YME1L shRNA (“aav-shYME1L-seq2”) or AAV-packed scramble control shRNA (“aav-shC”), the estimated pOS-1 xenograft volumes (**A**) and the nude mice body weights (**D**) were measured every 6 days. The daily pOS-1 xenograft growth, in mm^3^ per day, was estimated (**B**). All subcutaneous pOS-1 xenografts were isolated at Day-42 and weighted individually (**C**). Expression of listed mRNAs and proteins in the described pOS-1 xenograft tissues was tested (**E**, **F**, **I**, **J**, **K**). The TBAR activity (**G**) and ATP contents (**H**) in the described pOS-1 xenograft tissues were examined as well. The tissue sections were subject to to immunofluorescence staining of nuclear TUNEL (**L**). Data were presented as mean ± standard deviation (SD). *n* = 10 stands for 10 mice per group (**A**–**D**). **E**–**L** Five random tissues per xenograft were tested. *P < 0.001 versus “aav-shC” group. Scale bar = 100 μm.

Last, we tested the potential effect of YME1L on OS cell growth in vivo. To form OS xenografts, pOS-1 cells, at seven million cells in each mouse, were s.c. injected to the flanks of the nude mice. After 20 days, the pOS-1 xenograft-bearing mice were formed (“Day-0”, tumor volume close to 100 mm^3^) and were randomly assigned into two groups. The first treatment group xenograft mice received intratumoral injection AAV-packed YME1L shRNA (“aav-shYME1L-seq2”), whereas the second control group mice were intratumorally injected with AAV-packed scramble control shRNA (“aav-shC”). The virus were injected every 48 h (1.5 μL virus per xenograft, 1 × 10^9^ PFU) for a total of three time. The xenograft volumes were recorded every 6 days and were shown in Fig. 8A. Intratumoral injection of aav-shYME1L-seq2 robustly inhibited subcutaneous pOS-1 xenograft growth (Fig. 8A). The daily pOS-1 xenograft growth (in mm^3^ per day) was estimated, and results again showed that aav-shYME1L-seq2 injection hindered pOS-1 tumor growth (Fig. 8B). All subcutaneous pOS-1 xenografts were isolated at Day-42 and tumors with aav-shYME1L-seq2 injection were smaller and lighter than aav-shC-injected tumors (Fig. 8C). Among the two groups, the body weights were not significantly different (Fig. 8D). Thus, intratumoral injection of AAV-packed YME1L shRNA robustly suppressed subcutaneous pOS-1 xenograft growth in nude mice.

To explore the signaling changes by aav-shYME1L-seq2 in vivo, one pOS-1 xenograft per group was isolated carefully at “Day-18” and “Day-30”. A total of four pOS-1 xenografts were obtained. Half of the pOS-1 xenografts were cut into five pieces and tissue lysates were analyzed. As shown, expression of *YME1L* mRNA (Fig. 8E) and protein (Fig. 8F) was sharply decreased in aav-shYME1L-seq2-injected pOS-1 xenograft tissues. Supporting mitochondrial dysfunction and oxidative injury, we found that the TBAR activity was significantly strengthened in YME1L-silenced pOS-1 xenograft tissues (Fig. 8G), where ATP contents were decreased (Fig. 8H). Further tissue analyses revealed that phosphorylation of Akt (at Ser-473) and S6K (at Thr-389) was remarkably decreased in pOS-1 xenograft tissues with aav-shYME1L-seq2 injection (Fig. 8I, J), supporting that YME1L silencing inhibited Akt-mTOR activation in pOS-1 xenografts (Fig. 8I, J). The levels of cleaved-caspase-3 and cleaved-PAPR were robustly increased in aav-shYME1L-seq2-treated pOS-1 xenograft tissues (Fig. 8K). To further support apoptosis activation, we found that the percentage of TUNEL-positively stained nuclei was increased in aav-shYME1L-seq2-treated pOS-1 xenograft slides (Fig. 8L). These results together showed that YME1L silencing similarly induced mitochondrial dysfunction, oxidative injury, Akt-mTOR inactivation and apoptosis in pOS-1 xenografts.

## Discussion

Exploring novel diagnostic, prognostic, and predictive markers and developing targeted therapies are important for patients with advanced, recurrent or metastatic OS [5, 52–54]. Mitochondria in OS are actively involved in regulating energy metabolism, ROS balancing, calcium homeostasis, and cell death [40, 55, 56]. Dysregulation of mitochondria is a key contributor for oncogenesis and progression of OS [40, 55, 56]. Mitochondria-related cell death and biogenesis/metabolism are both dysregulated and intimately connected in OS [40]. Mitochondrial proteins, including POLRMT [17] and ADCK1 [16], are thereby proposed oncotargets of OS [40, 55, 56].

YME1L, locating in the mitochondrial inner membrane, is vital for the processing and degradation of various mitochondrial proteins [18]. It is also key in maintaining mitochondrial morphology, plasticity, mitochondrial protein import and metabolic activity [18]. MacVicar et al. have reported that mitochondrial reshaping by YME1L was required for pancreatic ductal adenocarcinoma (PDAC) cell growth in vitro and in vivo [19]. Overexpressed YME1L was also shown to be important for the growth of NSCLC cells and glioma cells [26, 27].

The results of the present study supported that YME1L could be an important therapeutic target of OS. *YME1L* expression is elevated in different human OS tissues and primary/immortalized OS cells. TCGA database shows that *YME1L* overexpression in sarcoma is correlated with poor overall survival and disease-specific survival of the patients. In primary/immortalized OS cells, YME1L silencing inhibited cell viability, proliferation, and migration, and resulted in cell cycle arrest and apoptosis. Whereas forced YME1L overexpression exerted pro-cancerous activity and strengthened OS cell growth. Importantly, intratumoral injection of YME1L shRNA AAV potently inhibited subcutaneous OS xenograft growth in nude mice. Thus, targeting the mitochondrial protein YME1L potently inhibited OS cell growth in vitro and in vivo.

It has been shown that YME1L is vital for maintaining mitochondrial hyperactivity and function in cancerous cells [26, 27]. Here found that YME1L silencing resulted in mitochondrial function impairment in OS cells, causing mitochondrial depolarization, ROS production, oxidative injury, lipid peroxidation and DNA damage as well as mito-complex I activity reduction and ATP depletion. Moreover, oxidative injury, lipid peroxidation and ATP reduction were detected in YME1L shRNA AAV-injected OS xenograft tissues. Therefore, mitochondrial dysfunction could be a key mechanism of YME1L silencing-induced anti-OS cell activity.

Hyperactivation of Akt-mTOR cascade is vital for the growth and progression of OS [42, 43, 57–59]. Zhu et al. showed that XL388, a mTORC1/2 dual inhibitor, induced cytotoxic, cytostatic and apoptotic activities in different OS cells [41]. Blocking Akt-mTOR activation by XL388 also arrested OS xenograft growth in nude mice [41]. NVP-BEZ235, a PI3K/mTOR dual inhibitor, inhibited proliferation and induced cell cycle arrest and apoptosis in different OS cells [60]. OS xenograft growth in nude mice was also suppressed by the dual inhibitor [60]. Yao et al. reported that the Akt inhibitor perifosine blocked Akt-mTOR activation, while activating caspase-3, c-Jun N-terminal kinases (JNK) and p53 cascades, eventually promoting OS cell apoptosis [61]. Bian et al. have shown that overexpressed Gαi3 immunoprecipitated with multiple receptor tyrosine kinases (VEGFR2, FGFR, EGFR, and etc.) to mediate downstream Akt-mTOR hyperactivation, thereby promoting OS cell in vitro and in vivo growth [32].

Recent studies have suggested that YME1L could be important for Akt-mTOR activation in cancer cells. Xia et al. have recently shown that Akt-mTOR activation in primary human NSCLC cell was decreased with YME1L silencing or KO, but was strengthened following YME1L overexpression [26]. Here, we found that YME1L is important for Akt-mTOR activation in OS cells. Phosphorylation of Akt and S6K was inhibited after YME1L silencing in primary OS cells, but was strengthened after YME1L overexpression. Restoring Akt-mTOR activation by S473D caAkt1 mitigated YME1L silencing-induced anti-OS cell activity. Akt-mTOR inactivation was also detected in YME1L shRNA AAV-treated OS xenografts. Thus, mediating Akt-mTOR activation could be a key mechanism of YME1L-induced OS cell growth.

Gαi proteins, including Gαi1 and Gαi3, were shown to associate with multiple ligand-activated RTKs, including EGFR [62], VEGFR [47], KGFR [50], TrkB [48] and c-Kit [39], essential for downstream Akt-mTOR cascade activation. Several non-RTK receptors also required Gαi1 and Gαi3 for activating the downstream Akt-mTOR cascade [38, 44, 46, 51]. Liu et al. discovered that YME1L overexpression in human glioma was vital for the expression of Gαi1 [27], thereby promoting downstream Akt-mTOR activation and cancer cell growth [27]. In our study, we discovered the crucial role of YME1L in regulating Gαi1 expression in OS cells as well. Silencing YME1L led to a decrease in both *Gαi1* mRNA and protein expression, whereas overexpressing YME1L resulted in increased Gαi1 levels. Notably, boosting Gαi1 expression through “oeGαi1” restored Akt-mTOR activation in YME1L-silenced glioma cells. This suggests that YME1L expression is essential for maintaining mitochondrial function and energy metabolism in OS cells, which in turn is necessary for the transcription and expression of Gαi1. Consequently, Gαi1 can act as a signaling mediator for activating the Akt-mTOR cascade through cell surface receptors. The underlying mechanism may warrant further characterizations.

The negligible impact of YME1L knockdown on both cell viability and caspase-3 activation within human osteoblasts might due to its low expression within osteoblasts. YME1L silencing in primary OS cells led to reduced phosphorylation of Akt and S6K, resulting in the inhibition of proliferation and migration, and induction of apoptosis. Importantly, our preliminary experiments have repeatedly found the basal activation level of Akt signaling in noncancerous human osteoblasts remained markedly low, potentially linked to the low expression of YME1L within these cells. This distinct dissimilarity underscores a possible explanation for the marginal influence observed on cell viability and caspase-3 activation in osteoblasts subsequent to YME1L depletion.

Treatment for advanced OS remains challenging [1, 4, 63]. Conventional chemotherapy often encounters resistance [64, 65], while aggressive surgical options may be limited in cases of metastasis or inoperable tumors [1, 4, 63]. The current research elucidates the significant functional role of YME1L overexpression in OS cell proliferation and progression. Highlighting YME1L’s localization to the inner mitochondrial membrane not only suggests a potential link between mitochondrial dysfunction and OS, but also sets the stage for exploring innovative therapeutic strategies targeting this protein. Although immediate clinical translation might be limited due to the absence of inhibitors targeting YME1L, this study lays a foundation for further research to develop inhibitors or explore alternative therapeutic avenues. It will offer promising prospects for future treatments aimed at mitigating OS progression and potentially enhancing broader cancer therapy approaches.

## Conclusion

Overexpressed YME1L promotes OS cell growth, possibly by maintaining mitochondrial function and Akt-mTOR activation.

### Supplementary information

Original data Set

Figure S1.

## Data Availability

All data generated during this study are included in this published article. Data will be made available upon request.
