# Supplementary figures and images for: Targeting the mitochondrial protein YME1L to inhibit osteosarcoma cell growth in vitro and in vivo
Source: Cell Death Dis. 2024 May 20;15(5):346. doi: 10.1038/s41419-024-06722-6 (PMC11106333; doi:10.1038/s41419-024-06722-6)

Figure S1: the uncropped blotting images

Figure 1

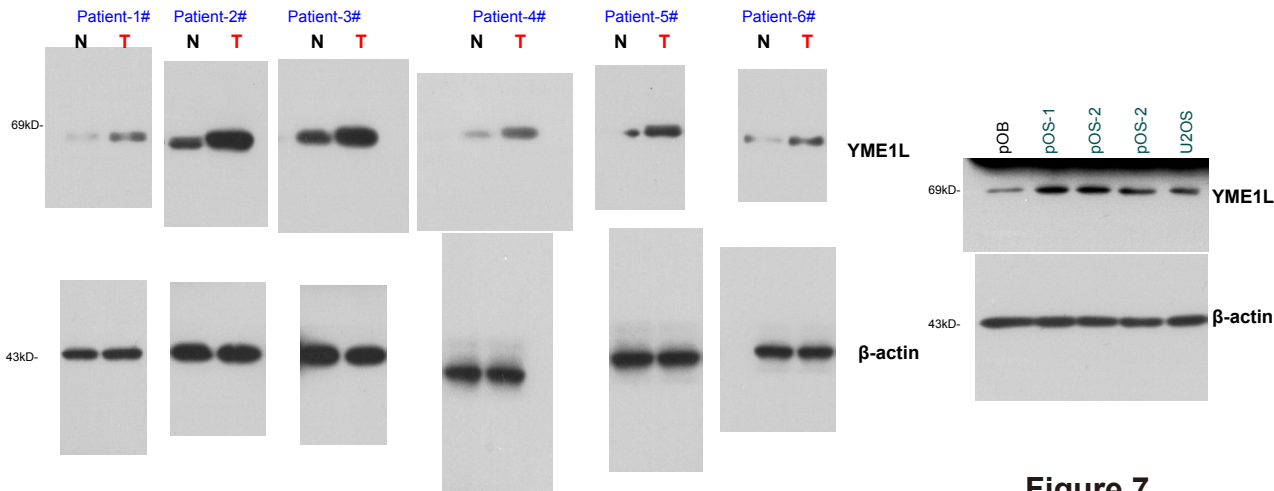

Figure 3

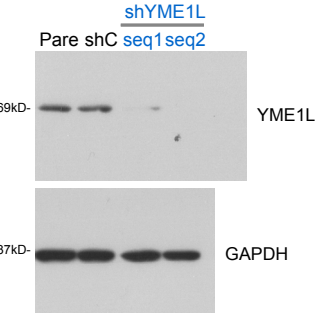

Figure 4

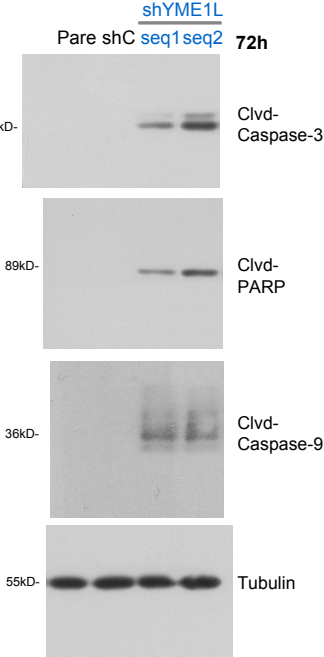

Figure 6

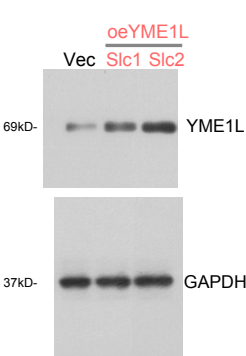

Figure 7

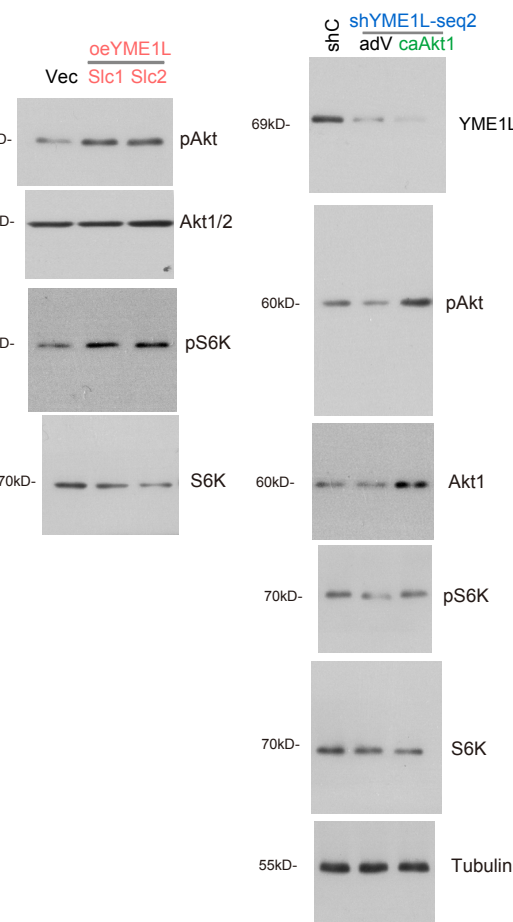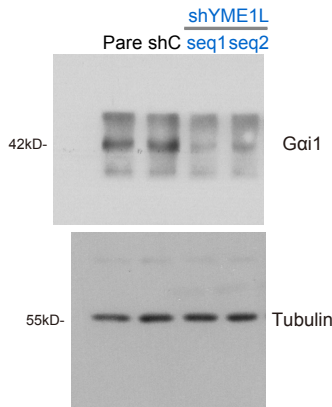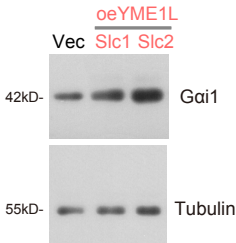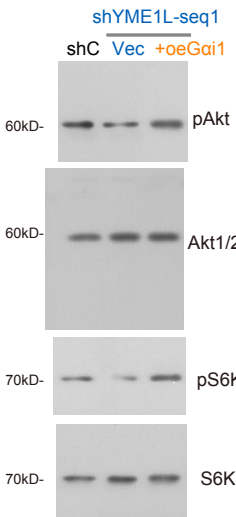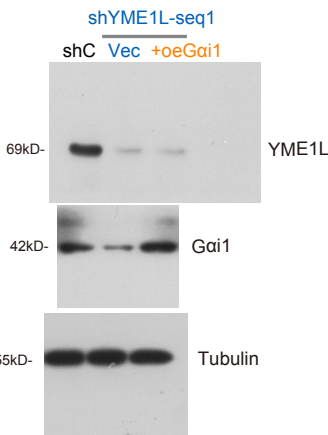

Figure 8.

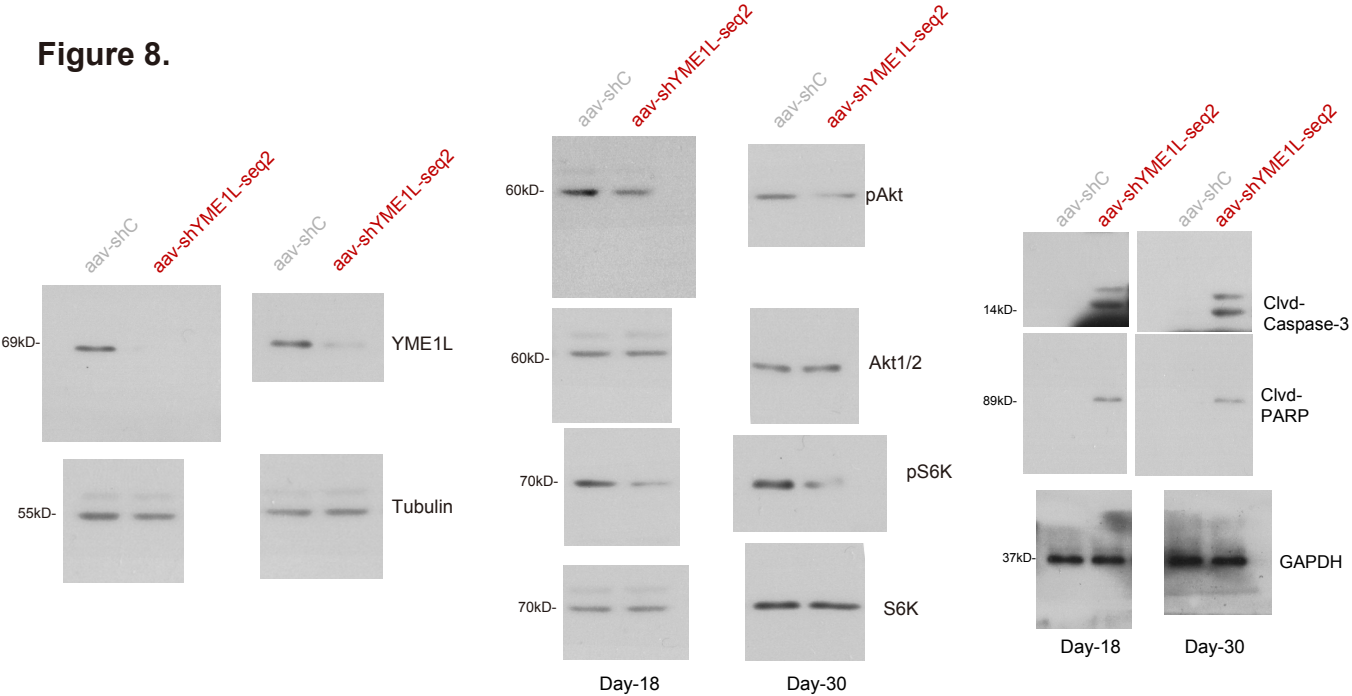

Supplement: Supplementary file 1 — Original data Set [file 41419_2024_6722_MOESM1_ESM.pdf]
